# Unique fixed point theorems in partially ordered metric spaces

**DOI:** 10.1016/j.heliyon.2020.e05563

**Published:** 2020-11-25

**Authors:** N. Seshagiri Rao, K. Kalyani

**Affiliations:** a Department of Applied Mathematics, School of Applied Natural Sciences, Adama Science and Technology University, Post Box No. 1888, Adama, Ethiopia; b Department of Mathematics, Vignan's Foundation for Science, Technology & Research, Vadlamudi-522213, Andhra Pradesh, India

**Keywords:** Mathematics, Partially ordered metric spaces, Rational contractions, Fixed point

## Abstract

In this paper, we establish some fixed point results of a mapping satisfying certain rational type contractive conditions in the frame work of a metric space endowed with partial order. Our results generalize and extend the result of Singh and Chatterjee (1988) [7] in partially ordered metric spaces and some existing results in the literature. Few illustrative examples are given to support our results.

## 1 Introduction

The Banach contraction principle is one of the most versatile result in fixed point theory and approximation theory. It plays an important role in solving many existing problems in pure and applied mathematics. There is a vast literature dealing with technical extensions and generalizations of Banach contraction principle, some instances of these works are in [1], [2], [3], [4], [5], [6], [7], [8], [9]. Besides, this famous classical theorem gives an iteration process through which we can obtain better approximation to the fixed point. It renders a key role in solving systems of linear algebraic equations involving iteration process. Iteration procedures are using in nearly every branch of applied mathematics, convergence proof and also in estimating the process of errors, very often by an application of Banach's fixed point theorem.

In recent times, fixed points of mappings in ordered metric spaces are of great use in many branches of mathematical analysis for solving nonlinear equations. The first result in this direction was initiated by Wolk [10] and later Monjardet [11] in partially order sets. Ran and Reurings [12] studied the existence of fixed points for certain mappings in partially ordered metric spaces and applied their results to matrix equations. The results of Ran and Reurings [12] were extended by Nieto et al. [13], [14], [15] for non decreasing mappings and obtained the solutions of certain partial differential equations with periodic boundary conditions. While Agarwal et al. [16] have discussed some new results for generalized contractions in partially ordered metric spaces. There have been a lot of generalizations and improvements of the results to obtain fixed point, common fixed point results for single valued and multivalued operators in various ordered spaces with topological properties, some of which are in [17], [18], [19], [20], [21], [22], [23], [24], [25], [26], [27], [28], [29], [30], [31], [32], [33], [34], [35], [36].

The purpose of this paper is to prove some fixed point results of a mapping satisfying nonlinear rational type contraction conditions in the context of a complete partially ordered metric space. These results generalize and extend few existing results of [7], [13], [22], [26] in the literature. We give some examples to show that our results are proper generalizations of the existing results.

## 2 Mathematical preliminaries

We start this section with the following frequently used definitions in our results.

Definition 1 [22] The triple (X,d,⪯) is called partially ordered metric space, if (X,⪯) is a partially ordered set together with (X,d) is a metric space.

Definition 2 [22] If (X,d) is a complete metric space, then triple (X,d,⪯) is called complete partially ordered metric space.

Definition 3 [22] A partially ordered metric space (X,d,⪯) is called ordered complete, if for each convergent sequence ${\left\{x_{n}\right\}}_{n=0}^{\infty }\subset X$, the following condition holds: either

• if $\left\{x_{n}\right\}$ is a non-decreasing sequence in *X* such that $x_{n}\to x$ implies $x_{n}⪯x$, for all n∈N that is, $x=\sup\{x_{n}\}$, or
• if $\left\{x_{n}\right\}$ is a non-increasing sequence in *X* such that $x_{n}\to x$ implies $x⪯x_{n}$, for all n∈N that is, $x=\inf\{x_{n}\}$.

Definition 4 Let (X,⪯) be a partially ordered set and let T:X→X be a mapping. Then

(1). elements x,y∈X are comparable, if x⪯y or y⪯x holds;
(2). a non empty set *X* is called well ordered set, if every two elements of it are comparable;
(3). *T* is said to be monotone nondecreasing w.r.t. ⪯, if for all x,y∈X,
  x⪯yimpliesTx⪯Ty;
(4). *T* is said to be monotone nonincreasing w.r.t. ⪯, if for all x,y∈X,
  x⪯yimpliesTx⪰Ty.

## 3 Main results

### 3.1 Results under generalized rational type contractions

In this section, we discuss the existence and uniqueness of a fixed point of a mapping satisfying nonlinear rational type contractive conditions in partially ordered metric spaces.

Theorem 1 *Let* (X,d,⪯) *be a complete partially ordered metric space. Suppose that a nondecreasing, continuous self mapping T on X satisfies the following condition:*

(1) $$d(Tx,Ty)\leq \left\{ \begin{array}{cc} \lambda d(x,y)+\theta \left[d(x,Tx)+d(x,Ty)\right]+\\ \;\;\;\;\;\mu \frac{d(x,Tx)d(x,Ty)+d(y,Tx)d(y,Ty)}{d(y,Tx)+d(x,Ty)} & ,\text{if}\;A\neq 0\\ 0 & ,\text{if}\;A=0 \end{array}\right.$$

*for all* x,y∈X *with* x⪯y*, where* A=d(y,Tx)+d(x,Ty) *and, there exist* λ,θ,μ∈[0,1) *such that* 0≤λ+2θ+μ<1*. If there exists a point* $x_{0}\in X$ *such that* $x_{0}⪯Tx_{0}$*, then T has a fixed point in X.*

Proof If $Tx_{0}=x_{0}$, then the proof is finished. Suppose that $x_{0}≺Tx_{0}$. Since *T* is nondecreasing mapping, we obtain by induction that

(2) $$x_{0}≺Tx_{0}=x_{1}⪯T^{2}x_{0}=x_{2}⪯...⪯T^{n}x_{0}=x_{n}⪯T^{n+1}x_{0}=x_{n+1}⪯...\;.$$

Thus, $\left\{x_{n}\right\}$ is a nondecreasing sequence in *X*. Put $x_{n+1}=Tx_{n}$. If there exists n≥1 such that $x_{n+1}=x_{n}$, then from $x_{n+1}=Tx_{n}=x_{n}$, $x_{n}$ is a fixed point and the proof is finished. Suppose that $x_{n+1}\neq x_{n}$ for all *n*. As the elements $x_{n}$ and $x_{n+1}$ are comparable for n≥1, we have the following cases:

**Case 1:** If $A=d(x_{n-1},Tx_{n})+d(x_{n},Tx_{n-1})\neq 0$, then from contraction condition (1), we have

$$\begin{array}{cc} d(x_{n+1},x_{n}) & =d(Tx_{n},Tx_{n-1})\\ & \leq \lambda d(x_{n},x_{n-1})+\theta \left[d(x_{n},Tx_{n})+d(x_{n},Tx_{n-1})\right]\\ & \;\;+\mu \frac{d(x_{n},Tx_{n})d(x_{n},Tx_{n-1})+d(x_{n-1},Tx_{n})d(x_{n-1},Tx_{n-1})}{d(x_{n-1},Tx_{n})+d(x_{n},Tx_{n-1})}, \end{array}$$

which intern implies that

$$\begin{array}{cc} d(x_{n+1},x_{n}) & \leq \lambda d(x_{n},x_{n-1})+\theta d(x_{n},x_{n+1})\\ & \;\;+\mu \frac{d(x_{n},x_{n+1})d(x_{n},x_{n})+d(x_{n-1},x_{n+1})d(x_{n-1},x_{n})}{d(x_{n-1},x_{n+1})+d(x_{n},x_{n})}. \end{array}$$

Finally, we arrive at

$$d(x_{n+1},x_{n})\leq {\left(\frac{\lambda +\mu }{1-\theta }\right)}^{n}d(x_{1},x_{0}).$$

Put $D=\frac{\lambda +\mu }{1-\theta }\in [0,1)$. Moreover, by triangular inequality for m≥n, we have

$$\begin{array}{cc} d(x_{m},x_{n})\leq & d(x_{m},x_{m-1})+d(x_{m-1},x_{m-2})+...+d(x_{n+1},x_{n})\\ \leq & \;\frac{D^{n}}{1-D}d(x_{1},x_{0}), \end{array}$$

as m,n→+∞, $d(x_{m},x_{n})\to 0$. So, $\left\{x_{n}\right\}$ is a Chachy sequence in a complete metric space *X*. Hence there exists u∈X such that

$$\lim_{n\to +\infty }x_{n}=u.$$

Further, the continuity of *T* implies that

$$Tu=T\left(\lim_{n\to +\infty }x_{n}\right)=\lim_{n\to +\infty }Tx_{n}=\lim_{n\to +\infty }x_{n+1}=u,$$

this proves that *u* is a fixed point of *T* in *X*.

**Case 2:** If $A=d(x_{n-1},Tx_{n})+d(x_{n},Tx_{n-1})=0$, then $d(x_{n+1},x_{n})=0$ from (1). This implies that $x_{n}=x_{n+1}$, a contradiction as the sequence points are comparable. Thus there exists a fixed point *u* of *T*. □

By relaxing the continuity criteria on *T* in Theorem 1, we have the following result.

Theorem 2 *Let* (X,d,⪯) *be a complete partially ordered metric space. Assume that X satisfies*

(3) $$\text{if a nondecreasing sequence}\;\{x_{n}\}\to x\;\text{in}\;X,\;\text{then}\;x=\sup\{x_{n}\}.$$

*Let* T:X→X *be a monotone nondecreasing mapping satisfying the contraction condition* (1)*. If there exists a point* $x_{0}\in X$ *with* $x_{0}⪯Tx_{0}$*, then T has a fixed point in X.*

Proof We only have to check that u=Tu. From the proof of Theorem 1, we have a nondecreasing sequence $\left\{x_{n}\right\}$ in *X* such that $x_{n}\to u\in X$. Thus, $u=\sup\{x_{n}\}$ for all n≥0 by (3). Since *T* is a nondecreasing mapping, then $Tx_{n}⪯Tu$ for all n∈N or, equivalently, $x_{n+1}⪯Tu$ for all n∈N. Further, $x_{0}≺x_{1}⪯Tu$ and $u=\sup\{x_{n}\}$, we get u⪯Tu.

Assume that u≺Tu. Using the similar argument as that in the proof of Theorem 1 for $x_{0}⪯Tx_{0}$, we obtain a nondecreasing sequence $\left\{T^{n}u\right\}$ in *X* such that $T^{n}u\to v\in X$. Again using (3), we get that $v=sup\{T^{n}u\}$. Moreover, from $x_{0}⪯u$, we obtain that $x_{n}=T^{n}x_{0}⪯T^{n}u$ for all n≥1 and $x_{n}≺T^{n}u$ for all n≥1 because $x_{n}⪯u≺Tu⪯T^{n}u$ for all n≥1.

As $x_{n}$ and $T^{n}u$ are comparable and distinct for n≥1, consider the following cases.

**Case 1:** Suppose that $d(T^{n}u,Tx_{n})+d(x_{n},T^{n+1}u)\neq 0$, then from contractive condition (1), we have

$$d(x_{n+1},T^{n+1}u)=d(Tx_{n},T(T^{n}u))\leq \lambda d(x_{n},T^{n}u)+\theta \left[d(x_{n},x_{n+1})+d(x_{n},T^{n+1}u)\right]\;\;+u\frac{d(x_{n},x_{n+1})d(x_{n},T^{n+1}u)+d(T^{n}u,x_{n+1})d(T^{n}u,T^{n+1}u)}{d(T^{n}u,x_{n+1})+d(x_{n},T^{n+1}u)}.$$

On taking n→+∞ in the above inequality, we obtain that

d(u,v)≤(λ+θ)d(u,v).

As λ+θ<1, we get that d(u,v)=0, thus u=v. Particularly, $u=v=sup\{T^{n}u\}$ and consequently, Tu⪯u, which contradicts the fact that Tu≺u. Hence, we conclude that Tu=u.

**Case 2:** If $d(T^{n}u,Tx_{n})+d(x_{n},T^{n+1}u)=0$, then $d(x_{n+1},T^{n+1}u)=0$. Letting n→+∞, we obtain that d(u,v)=0. Then $u=v=sup\{T^{n}u\}$, which implies that Tu⪯u, a contradiction again. Therefore, Tu=u. □

Now, we present some examples where it can be appreciated that hypotheses in Theorem 1 and Theorem 2 do not guarantee uniqueness of the fixed point. These examples appear in [13].

Example 1 Let $X=\{(1,0),(0,1)\}\subseteq R^{2}$ with the Euclidean distance *d*. We consider the partial order *U* in *X* as follows:

U:(u,v)≤(r,s)if and only ifu≤randv≤s.

Let T:X→X by T(x,y)=(x,y). Then *T* have fixed points in *X*.

Proof It is clear that (X,d,≤) is a complete partially ordered metric space. Besides, the identity mapping T(x,y)=(x,y) is trivially continuous and nondecreasing and satisfying the contraction condition

$$d(T(u,v),T(r,s))\;\leq \lambda d((u,v),(r,s))\;\leq \lambda d((u,v),(r,s))+\theta \left[d((u,v),T(u,v))+d((u,v),T(r,s))\right]\;\;\;+\mu \frac{d((u,v),T(u,v))d((u,v),T(r,s))+d((r,s),T(u,v))d((r,s),T(r,s))}{d((r,s),T(u,v))+d((u,v),T(r,s))}$$

for any λ,θ,μ∈[0,1) with 0≤λ+2θ+μ<1. Notice that the elements of *X* are only comparable to themselves. Moreover, (1,0)≤T((1,0)). Here all the conditions of Theorem 1 are satisfied and *T* has two fixed points, which are (1,0) and (0,1). □

Example 2 Under the same assumptions in Example 1, let us consider a nondecreasing sequence $\{(x_{n},y_{n})\}\subseteq X$ converging to (x,y). Then necessarily, $\left\{(x_{n},y_{n})\right\}$ is a constant sequence and $\left(x_{n},y_{n})=(x,y\right)$ for all n∈N. Also, note that the limit (x,y) is an upper bound, of course supreme for all the terms of the sequence. Hence, all the conditions of Theorem 2 are satisfied and, (1,0) and (0,1) are two fixed points of *T* in *X*.

Now we give a sufficient condition for the uniqueness of the fixed point that exists in Theorem 1 and Theorem 2.

(4) every pair of elements has a lower bound or an upper bound.

In [13], it is proved that the above mentioned condition is equivalent to

for everyx,y∈X,there existsϑ∈Xwhich is comparable toxandy.

Theorem 3 *In addition to the hypotheses of* Theorem 1 *(or* Theorem 2*), condition* (4) *provides uniqueness of the fixed point of T in X.*

Proof Suppose that there exists x,y∈X are fixed points of *T*.

We distinguish two cases.

**Case 1:** Suppose *x* and *y* are comparable and distinct. Now, we have the following two subcases:

(i). If d(y,Tx)+d(x,Ty)≠0 then from (1), we have

$$d(x,y)=d(Tx,Ty)\leq \lambda d(x,y)+\theta \left[d(x,Tx)+d(x,Ty)\right]+\mu \frac{d(x,Tx)\;d(x,Ty)+d(y,Tx)\;d(y,Ty)}{d(y,Tx)+d(x,Ty)}\leq \lambda d(x,y)+\theta \left[d(x,x)+d(x,y)\right]+\mu \frac{d(x,x)\;d(x,y)+d(y,x)\;d(y,y)}{d(y,x)+d(x,y)}\leq \left(\lambda +\theta \right)d(x,y)<\;d(x,y),\;\text{since}\;\lambda +\theta <1,$$

which is a contradiction. Therefore, x=y.

(ii). If d(y,Tx)+d(x,Ty)=0, then d(x,y)=0, a contradiction again. Hence, x=y.

**Case 2:** If *x* and *y* are not comparable, then by condition (4) there exists ϑ∈X comparable to *x* and *y*. Monotonicity implies that $T^{n}\vartheta$ is comparable to $T^{n}x=x$ and $T^{n}y=y$ for all n≥0.

Suppose that $T^{m}\vartheta =x$, for some m≥1, then as *x* is a fixed point, the sequence $\left\{T^{n}\vartheta :n\geq m\right\}$ is constant and consequently $T^{n}\vartheta \to x$ as n→+∞. On the other hand, if $T^{n}\vartheta \neq x$ for all n≥1. Now, again we have the following two subcases:

(i). If $d(T^{n-1}x,T^{n}\vartheta )+d(T^{n-1}\vartheta ,T^{n}x)\neq 0$, then using the contractive condition (1) for n≥2, we obtain that

$$\begin{array}{cc} d(T^{n}\vartheta ,x) & =d(T^{n}\vartheta ,T^{n}x)\\ & \leq \lambda d(T^{n-1}\vartheta ,T^{n-1}x)+\theta \left[d(T^{n-1}\vartheta ,T^{n}\vartheta )+d(T^{n-1}\vartheta ,T^{n}x)\right]\\ & \;\;+\mu \frac{d(T^{n-1}\vartheta ,T^{n}\vartheta )\;d(T^{n-1}\vartheta ,x)+d(x,T^{n}\vartheta )\;d(x,x)}{d(T^{n}\vartheta ,x)+d(x,T^{n-1}\vartheta )}\\ & \leq \lambda d(T^{n-1}\vartheta ,x)+\theta \left[d(T^{n-1}\vartheta ,x)+d(T^{n}\vartheta ,x)\right]+\mu d(T^{n-1}\vartheta ,x). \end{array}$$

This implies that

$$d(T^{n}\vartheta ,x)\leq \left(\frac{\lambda +\theta +\mu }{1-\theta }\right)d(T^{n-1}\vartheta ,x).$$

Inductively, we obtain that

$$d(T^{n}\vartheta ,x)\leq {\left(\frac{\lambda +\theta +\mu }{1-\theta }\right)}^{n}d(\vartheta ,x).$$

As 0≤λ+2θ+μ<1 and letting n→+∞ in the above inequality, we get

$$\lim_{n\to +\infty }T^{n}\vartheta =x.$$

By following the similar argument as above, we can prove that

$$\lim_{n\to +\infty }T^{n}\vartheta =y.$$

Therefore from uniqueness of a limit, we have x=y.

(ii). If $d(T^{n-1}x,T^{n}\vartheta )+d(T^{n-1}\vartheta ,T^{n}x)=0$, then by contraction condition (1), we have $d(T^{n}\vartheta ,x)=0$. Therefore,

$$\lim_{n\to +\infty }T^{n}\vartheta =x.$$

By similar argument, we can prove that

$$\lim_{n\to +\infty }T^{n}\vartheta =y.$$

Finally, the uniqueness of the limit implies that x=y. Hence, *T* has a unique fixed point in *X*. □

Example 3 It is easily proved that the space C[0,1]={x:[0,1]→R,continuous} with the partial order given by

x≤yif and only ifx(t)≤y(t),fort∈[0,1],

and the metric given by

d(x,y)=sup⁡{|x(t)−y(t)|:t∈[0,1]},

satisfies condition (3). Moreover, as for x,y∈[0,1], the function max⁡(x,y)(t)=max⁡{x(t),y(t)} is continuous. Also (C[0,1],≤) satisfies the condition (4).

Example 4 Let $X=\{(0,0),(\frac{1}{2},0),(0,1)\}$ be a subset of $R^{2}$ with the order ≤ defined as: for $(x_{1},y_{1}),(x_{2},y_{2})\in X$ with $\left(x_{1},y_{1})\leq (x_{2},y_{2}\right)$ if and only if $x_{1}\leq x_{2}$ and $y_{1}\leq y_{2}$. Let the distance d:X×X→R is defined by

$$d((x_{1},y_{1}),(x_{2},y_{2}))=\max\{|x_{1}-x_{2}|,|y_{1}-y_{2}|\}.$$

Let T:X→X be defined by T(0,0)=(0,0), $T(0,1)=(\frac{1}{2},0)$ and $T(\frac{1}{2},0)=(0,0)$. Here all the conditions of Theorem 1, Theorem 2, Theorem 3 are satisfied and (0,0) is the unique fixed point of *T*.

Note 1

(i). If θ=μ=0 in Theorem 1, Theorem 2, Theorem 3, then we obtain Theorems 2.1, 2.2 and 2.3 of [13].
(ii). If θ=0 in Theorem 1, Theorem 2, Theorem 3, then we get Theorems 15, 17 and 18 of [22].
(iii). If λ=θ=0 in Theorem 1, Theorem 2, we obtain Theorem 20 of [22]. Also, the uniqueness of the fixed point can be proved by using Condition (4) in the hypotheses.

If in the Theorem 1, Theorem 2, λ=0, we obtain the following fixed point theorem in complete partially ordered metric spaces.

Theorem 4 *Let* (X,d,⪯) *be a complete partially ordered metric space. Suppose that a nondecreasing self mapping T on X satisfies the following condition:*

(5) $$d(Tx,Ty)\leq \left\{ \begin{array}{c} \theta \left[d(x,Tx)+d(x,Ty)\right]+\mu \frac{d(x,Tx)d(x,Ty)+d(y,Tx)d(y,Ty)}{d(y,Tx)+d(x,Ty)}\;,\text{if}\;A\neq 0\\ 0\;\;\;\;\;\;\;\;\;\;\;\;\;\;\;\;\;\;\;\;\;\;\;\;\;\;\;\;\;\;\;\;\;\;\;\;\;\;\;\;\;\;\;\;\;\;\;\;\;\;\;\;\;\;\;\;\;\;\;\;\;\;\;\;\;\;\;\;\;\;\;\;,\text{if}\;A=0 \end{array}\right.$$

*for all* x,y∈X *with* x⪯y*, where* A=d(y,Tx)+d(x,Ty) *and, there exist* θ,μ∈[0,1) *such that* 0≤2θ+μ<1*. And also suppose that either T is continuous or X satisfies condition* (3)*. If there exists* $x_{0}\in X$ *such that* $x_{0}⪯Tx_{0}$*, then T has a fixed point in X.*

Theorem 5 *The uniqueness of the fixed point in* Theorem 4 *can be proved using Condition* (4)*.*

Theorem 6 *Let* (X,d,⪯) *be a complete partially ordered metric space. Assume that either T is continuous or X is such that*

$$\text{if a nonincreasing sequence}\;\{x_{n}\}\to x\;\text{in}\;X,\;\text{then}\;x=\inf\{x_{n}\}.$$

*Let* T:X→X *be a monotone nondecreasing mapping satisfying the contraction condition* (1) *(or* (5)*). If there exists* $x_{0}\in X$ *with* $x_{0}⪰Tx_{0}$*, then T has a fixed point in X.*

Proof The scheme of the proof is similar to the procedure followed in the proof of the previous Theorem 1, Theorem 2. □

Theorem 7 *Condition* (4) *provides uniqueness of the fixed point of T in the hypotheses of* Theorem 6*.*

### 3.2 Results under Singh and Chatterjee contractions

We begin this section with the following definition.

Definition 5 Let (X,d,⪯) be a partially ordered metric space. A mapping T:X→X is called an almost Singh and Chatterjee contraction, if there exist α,β,γ∈[0,1) with 0≤α+2β+γ<1 and L≥0 such that

(6) $$\begin{array}{cc} d(Tx,Ty) & \leq \alpha \frac{d(x,Tx)\;d(y,Ty)}{d(x,y)}+\beta [d(x,Ty)+d(y,Tx)]\\ & \;\;+\gamma d(x,y)+L\;\min\{d(x,Ty),d(y,Tx),d(x,Tx)\}, \end{array}$$

for all distinct x,y∈X with x⪯y.

Now, we establish some results for the existence of a fixed point of a mapping satisfying an almost Singh and Chatterjee contraction in the setup of partially ordered metric spaces. The first result in this subsection is the following fixed point theorem.

Theorem 8 *Suppose that* (X,d,⪯) *be a complete partially ordered metric space. Let* T:X→X *be an almost Singh and Chatterjee contraction and, T is nondecreasing and continuous. If there exists* $x_{0}\in X$ *such that* $x_{0}⪯Tx_{0}$*, then T has a fixed point in X.*

Proof For $x_{0}\in X$, set a sequence $\{x_{n}\}\subseteq X$ by $x_{n+1}=Tx_{n}$ for n≥0. If $Tx_{n_{0}}=x_{n_{0}}$ for some $n_{0}\in N$, then the proof is finished. Suppose that $x_{n}\neq x_{n+1}$ for all *n*. Since $x_{0}⪯Tx_{0}$ and *T* is nondecreasing, then by induction we obtain that

(7) $$x_{0}⪯x_{1}⪯...⪯x_{n}⪯x_{n+1}⪯...\;.$$

Thus, $\left\{x_{n}\right\}$ is a nondecreasing sequence and the elements of the sequence are comparable.

Now,

$$\begin{array}{cc} d(x_{n+1},x_{n}) & =d(Tx_{n},Tx_{n-1})\\ & \leq \alpha \frac{d(x_{n},Tx_{n})d(x_{n-1},Tx_{n-1})}{d(x_{n},x_{n-1})}+\beta [d(x_{n},Tx_{n-1})+d(x_{n-1},Tx_{n})]\\ & \;\;+\gamma d(x_{n},x_{n-1})+L\;\min\{d(x_{n},Tx_{n-1}),d(x_{n-1},Tx_{n}),d(x_{n},Tx_{n})\}, \end{array}$$

which implies that

$$d(x_{n+1},x_{n})\leq \left(\frac{\beta +\gamma }{1-\alpha -\beta }\right)d(x_{n},x_{n-1})\leq ...\leq {\left(\frac{\beta +\gamma }{1-\alpha -\beta }\right)}^{n}d(x_{1},x_{0}).$$

Therefore by the triangular inequality for m≥n, we have

$$\begin{array}{cc} d(x_{n},x_{m})= & d(x_{n},x_{n+1})+d(x_{n+1},x_{n+2})+...+d(x_{m-1},x_{m})\\ \leq & \;\left(s^{n}+s^{n+1}+...+s^{m-1}\right)d(x_{0},Tx_{0})\\ \leq & \;\frac{s^{n}}{1-s}\;d(x_{1},x_{0}), \end{array}$$

where $s=\frac{\beta +\gamma }{1-\alpha -\beta }\in [0,1)$. Taking limit as m,n→+∞ in the above inequality, we obtain that $d(x_{n},x_{m})=0$. Thus, $\left\{x_{n}\right\}$ is a Cauchy sequence in *X*. Since *X* is a complete metric space, then there exists u∈X such that

$$\lim_{n\to +\infty }x_{n}=u.$$

From the continuity of *T*, we have

$$Tu=T\left(\lim_{n\to +\infty }x_{n}\right)=\lim_{n\to +\infty }Tx_{n}=\lim_{n\to +\infty }x_{n+1}=u.$$

Hence, *u* is a fixed point of *T* in *X*. □

Theorem 9 *Let* (X,d,⪯) *be a complete partially ordered metric space. Assume that X satisfies*

(8) $$\text{if a nondecreasing sequence}\;\{x_{n}\}\to x\;\text{in}\;X,\;\text{then}\;x=\sup\{x_{n}\}.$$

*Let* T:X→X *be a monotone nondecreasing mapping satisfying the contraction condition* (6)*. If there exists* $x_{0}\in X$ *with* $x_{0}⪯Tx_{0}$*, then T has a fixed point in X.*

Proof The proof follows Theorem 2. □

Now, we give the examples for Theorem 8.

Example 5 Let $X=\{(2,0),(0,2)\}\subseteq R^{2}$ with the Euclidean distance *d*. We consider the partial order in *X* as follows:

$$(x_{1},y_{1})\leq (x_{2},y_{2})\;\text{if and only if}\;x_{1}\leq x_{2}\;\text{and}\;y_{1}\leq y_{2}.$$

Thus, (X,d,≤) is a complete partially ordered metric space. The mapping T(x,y)=(x,y) is continuous, nondecreasing and the contraction condition

$$d(T(x_{1},y_{1}),T(x_{2},y_{2}))\;\leq \gamma d((x_{1},y_{1}),(x_{2},y_{2}))\;\leq \alpha \frac{d((x_{1},y_{1}),T(x_{1},y_{1}))d((x_{2},y_{2}),T(x_{2},y_{2}))}{d((x_{1},y_{1}),(x_{2},y_{2}))}\;\;+\beta \left[d((x_{1},y_{1}),T(x_{2},y_{2}))+d((x_{2},y_{2}),T(x_{1},y_{1}))\right]+\gamma d((x_{1},y_{1}),(x_{2},y_{2}))\;+L\min\{d((x_{1},y_{1}),T(x_{2},y_{2})),d((x_{2},y_{2}),T(x_{1},y_{1})),d((x_{1},y_{1}),T(x_{1},y_{1}))\}$$

holds for any α,β,γ∈[0,1) with 0≤α+2β+γ<1 and for any L≥0. Notice that the elements of *X* are only comparable to themselves and no different elements are comparable. Moreover, (0,2)≤T((0,2)). Here all the conditions of Theorem 8 are satisfied, (2,0) and (0,2) are the fixed points of *T*.

Example 6 Let X={(x,−x),x∈R} with usual order and *d* be the Euclidean distance. The identity map has an infinite number of fixed points in *X*. Note that two different points in $X_{2}$ are not comparable.

Theorem 10 *In addition to the hypotheses of* Theorem 8 *(or* Theorem 9*), condition* (4) *provides uniqueness of the fixed point of T in X.*

Proof The proof follows Theorem 3. □

Now, we illustrate an example for Theorem 10.

Example 7 Let X=[0,1] with usual metric and usual order ≤. We define an operator T:X→X as follows:

$$T(x)=\left\{ \begin{array}{cc} \;\frac{x}{24} & ,\text{if}\;x\in [0,\frac{1}{4}]\\ \frac{x}{12}-\frac{1}{96} & ,\text{if}\;x\in (\frac{1}{4},1]. \end{array}\right.$$

Then *T* has a unique fixed point in *X*.

Proof The mapping *T* is continuous and nondecreasing and, let $x_{0}=0$ then $x_{0}\leq Tx_{0}$. Note that any two different points are comparable in *X*. Take $\gamma =\frac{1}{10}$. Then for any α,β∈[0,1) with α+2β+γ<1, we have the result. Let us examine in detail. Without loss of generality, we assume that y⪯x.

**Case 1:** If $x,y\in [0,\frac{1}{4})$, then

$$\begin{array}{cc} d(Tx,Ty)= & \frac{1}{24}|x-y|\leq \frac{1}{10}|x-y|=\frac{1}{10}\;d(x,y)\\ & \leq \frac{d(x,Tx)d(y,Ty)}{d(x,y)}+\beta [d(x,Ty)+d(y,Tx)]\\ & \;\;+\frac{1}{10}d(x,y)+L\;\min\{d(x,Ty),d(y,Tx),d(x,Tx)\}, \end{array}$$

holds for any L≥0 and for α,β∈[0,1) with α+2β+γ<1. Thus all conditions of Theorem 8 are satisfied.

**Case 2:** Suppose $x,y\in (\frac{1}{4},1]$, then

$$\begin{array}{cc} d(Tx,Ty) & =\frac{1}{12}|x-y|\leq \frac{1}{10}|x-y|=\frac{1}{10}\;d(x,y)\\ & \leq \frac{d(x,Tx)d(y,Ty)}{d(x,y)}+\beta [d(x,Ty)+d(y,Tx)]\\ & \;\;+\frac{1}{10}d(x,y)+L\;\min\{d(x,Ty),d(y,Tx),d(x,Tx)\}, \end{array}$$

holds for any L≥0 and for some α,β∈[0,1) with α+2β+γ<1. Hence, all conditions of Theorem 8 are satisfied.

**Case 3:** If $y\in [0,\frac{1}{4})\;\text{and}\;x\in (\frac{1}{4},1]$, then we can easily evaluate that $\frac{1}{96}|4x-1|\leq \frac{1}{96}$. Further, we have $\frac{23}{96}\leq d(x,Ty)=|x-\frac{y}{24}|\leq 1$, $\frac{1}{96}\leq d(y,Tx)=|\frac{x}{12}-\frac{1}{96}-y|\leq \frac{23}{96}$ and $\frac{23}{96}\leq d(x,Tx)=|\frac{x}{12}-\frac{1}{96}-y|\leq \frac{89}{96}$. By the help of these observations, we derive that

$$\begin{array}{cc} d(Tx,Ty) & =\left|\frac{x}{12}-\frac{1}{96}-\frac{y}{24}\right|\\ & \leq \frac{1}{24}|x-y|+\frac{1}{96}|4x-1|\\ & \leq \frac{d(x,Tx)d(y,Ty)}{d(x,y)}+\beta [d(x,Ty)+d(y,Tx)]\\ & \;\;+\frac{1}{10}d(x,y)+L\;\min\{d(x,Ty),d(y,Tx),d(x,Tx)\}, \end{array}$$

holds for any L≥0 and for α,β∈[0,1) with α+2β+γ<1. Thus all conditions of Theorem 8 and Condition (4) are satisfied in *X*. Therefore, 0∈X is the unique fixed point of *T*. □

Definition 6 Let (X,d,⪯) be a partially ordered metric space. A self-mapping *T* on *X* is called a Singh and Chatterjee contraction, if there exist α,β,γ∈[0,1) with 0≤α+2β+γ<1 such that

(9) $$d(Tx,Ty)\leq \alpha \frac{d(x,Tx)\;d(y,Ty)}{d(x,y)}+\beta [d(x,Ty)+d(y,Tx)]+\gamma d(x,y),$$

for all distinct x,y∈X with x⪯y.

Corollary 1 *Suppose that* (X,d,⪯) *be a complete partially ordered metric space. Let* T:X→X *be a Singh and Chatterjee contraction, nondecreasing and continuous. If there exists* $x_{0}\in X$ *such that* $x_{0}⪯Tx_{0}$*, then T has a fixed point in X.*

Proof Set L=0 in Theorem 8. □

Besides, if *X* satisfies the condition (3), then a map *T* has a fixed point and also, if *X* satisfies condition (4), then one obtains uniqueness of the fixed point.

Theorem 11 *Let* (X,d,⪯) *be a complete partially ordered metric space. Assume that either T is continuous or X is such that*

$$\text{if a nonincreasing sequence}\;\{x_{n}\}\to x\;\text{in}\;X,\;\text{then}\;x=\inf\{x_{n}\}.$$

*Let* T:X→X *be a monotone nondecreasing mapping satisfying the contraction condition* (6) *(or* (9)*). If there exists* $x_{0}\in X$ *with* $x_{0}⪰Tx_{0}$*, then T has a fixed point in X.*

Proof The scheme of the proof is similar to the procedure followed in the proof of the previous theorems. □

We present an example where Theorem 8 (or Corollary 1) can be applied and this example cannot be treated by the main theorem of Singh and Chatterjee [7] in complete metric space.

Example 8 Let X={(0,1),(1,0),(1,1)} and consider the partial order relation on *X* by R={(x,x):x∈X}. Notice that elements in *X* are only comparable to themselves. Besides, (X,d) is a complete metric space, where *d* is an Euclidean distance. Also, (X,≤) is a partially ordered set.

Let T:X→X be defined by

T(0,1)=(1,0),T(1,0)=(0,1),T(1,1)=(1,1).

Thus, *T* is trivially continuous and nondecreasing and satisfy the condition (6) (or condition (9)) of Theorem 8 (or Corollary 1), since elements of *X* are only comparable to themselves. Moreover, (1,1)≤T(1,1)=(1,1) and, by Theorem 8 (or Corollary 1), *T* has fixed point (1,1).

On the other hand, for x=(0,1), y=(1,0) in *X*, we have

$$d(Tx,Ty)=\sqrt{2},\;d(x,Ty)=0,\;d(y,Tx)=0,\;d(x,Tx)=\sqrt{2},\;d(y,Ty)=\sqrt{2},$$

and the contractive condition of the main theorem of Singh and Chatterjee [7] is not satisfied because

(10) $$\begin{array}{cc} d(Tx,Ty)=\sqrt{2} & \leq \alpha \frac{d(x,Tx)\;d(y,Ty)}{d(x,y)}+\beta [d(x,Ty)+d(y,Tx)]+\gamma d(x,y)\\ & \leq \alpha .\frac{\sqrt{2}.\sqrt{2}}{\sqrt{2}}+\beta .0+\gamma .\sqrt{2}\\ & =(\alpha +\gamma ).\sqrt{2} \end{array}$$

and thus, α+γ≥1. Consequently, this example can not be treated by the main theorem of Singh and Chatterjee [7].

Moreover, notice that in this example we have uniqueness of fixed point and (X,≤) does not satisfy condition (4). This proves that condition (4) is not necessary condition for the uniqueness of the fixed point.

Now, in the next theorem we establish a fixed point of a self mapping *T* by assuming only the continuity of some iteration of *T*.

Theorem 12 *Let* (X,d,⪯) *be a complete partially ordered metric space. Suppose that* T:X→X *be an almost Singh and Chatterjee contraction and nondecreasing. Also, suppose there exists* $x_{0}\in X$ *such that* $x_{0}⪯Tx_{0}$*. If the mapping* $T^{r}$ *is continuous for some positive integer r, then T has a fixed point in X.*

Proof From the proof of Theorem 8, we construct a nondecreasing sequence $\left\{x_{n}\right\}$ in *X* such that $x_{n}\to \mu$, for some μ∈X. Also, its subsequence $x_{n_{k}}(n_{k}=kr)$ converges to the same point *μ*.

Therefore,

$$T^{r}\mu =T^{r}\left(\lim_{n\to +\infty }x_{n_{k}}\right)=\lim_{n\to +\infty }x_{n_{k+1}}=\mu .$$

Thus, *μ* is a fixed point of $T^{r}$.

Next to prove that *μ* is a fixed point of *T*. Let *m* be the least positive integer such that $T^{m}\mu =\mu$ and $T^{q}\mu \neq \mu \;(q=1,2,3,....,m-1)$. If m>1, then

$$\begin{array}{cc} d(T\mu ,\mu ) & =d(T\mu ,T^{m}\mu )\\ & \leq \alpha \frac{d(\mu ,T\mu )\;d(T^{m-1}\mu ,T^{m}\mu )}{d(\mu ,T^{m-1}\mu )}+\beta [d(\mu ,T^{m}\mu )+d(T^{m-1}\mu ,T\mu )]\\ & \;\;+\gamma d(\mu ,T^{m-1}\mu )+L\;\min\{d(\mu ,T^{m}\mu ),d(T^{m-1}\mu ,T\mu ),d(\mu ,T\mu )\}, \end{array}$$

which implies that

$$d(\mu ,T\mu )\leq \left(\frac{\beta +\gamma }{1-\alpha -\beta }\right)d(\mu ,T^{m-1}\mu ).$$

Again from contraction condition (6), we have

$$\begin{array}{cc} d(\mu ,T^{m-1}\mu ) & =d(T^{m}\mu ,T^{m-1}\mu )\\ & \leq \alpha \frac{d(T^{m-1}\mu ,T^{m}\mu )\;d(T^{m-2}\mu ,T^{m-1}\mu )}{d(T^{m-1}\mu ,T^{m-2}\mu )}\\ & +\beta [d(T^{m-1}\mu ,T^{m-1}\mu )+d(T^{m-2}\mu ,T^{m}\mu )]+\gamma d(T^{m-1}\mu ,T^{m-2}\mu )\\ & +L\;\min\{d(T^{m-1}\mu ,T^{m-1}\mu ),d(T^{m-2}\mu ,T^{m}\mu ),d(T^{m-1}\mu ,T^{m}\mu )\}. \end{array}$$

Inductively, we get

$$d(\mu ,T^{m-1}\mu )=d(T^{m}\mu ,T^{m-1}\mu )\leq sd(T^{m-1}\mu ,T^{m-2}\mu )\leq ...\leq s^{m-1}d(T\mu ,\mu ),$$

where $s=\frac{\beta +\gamma }{1-\alpha -\beta }\in [0,1)$. Therefore,

$$d(T\mu ,\mu )\leq s^{m}d(T\mu ,\mu )<d(T\mu ,\mu ),$$

which is a contradiction. Hence, Tμ=μ. □

Corollary 2 *Let* (X,d,⪯) *be a complete partially ordered metric space. Suppose that* T:X→X *be a Singh and Chatterjee contraction and nondecreasing. Suppose there exists* $x_{0}\in X$ *such that* $x_{0}⪯Tx_{0}$*. If the mapping* $T^{r}$ *is continuous for some positive integer r, then T has a fixed point in X.*

Proof Set L=0 in Theorem 12, we obtain the required proof. □

Theorem 13 *Let* (X,d,⪯) *be a complete partially ordered metric space and let T be a nondecreasing self mapping defined on X. Suppose that for some positive integer m, the self mapping T satisfies the following condition:*

(11) $$\begin{array}{cc} d(T^{m}x,T^{m}y) & \leq \alpha \frac{d(x,T^{m}x)\;d(y,T^{m}y)}{d(x,y)}+\beta [d(x,T^{m}y)+d(y,T^{m}x)]+\gamma d(x,y)\\ & \;\;+L\;\min\{d(x,Ty),d(y,Tx),d(x,Tx)\}, \end{array}$$

*for all distinct* x,y∈X *with* x⪯y*, where* α,β,γ∈[0,1) *with* 0≤α+2β+γ<1 *and* L≥0*. Suppose there exists* $x_{0}\in X$ *such that* $x_{0}⪯T^{m}x_{0}$*. If* $T^{m}$ *is continuous, then T has a fixed point in X.*

Proof The proof follows from Theorem 8 and Theorem 12. □

Corollary 3 *Let* (X,d,⪯) *be a complete partially ordered metric space and let T be a nondecreasing self mapping defined on X. Suppose that for some positive integer m, the self mapping T satisfies the following condition:*

(12) $$d(T^{m}x,T^{m}y)\leq \alpha \frac{d(x,T^{m}x)\;d(y,T^{m}y)}{d(x,y)}+\beta [d(x,T^{m}y)+d(y,T^{m}x)]+\gamma d(x,y),$$

*for all distinct* x,y∈X *with* x⪯y*, where* α,β,γ∈[0,1) *with* 0≤α+2β+γ<1*. Suppose there exists* $x_{0}\in X$ *with* $x_{0}⪯T^{m}x_{0}$*. If* $T^{m}$ *is continuous, then T has a fixed point in X.*

Proof Set L=0 in Theorem 13. □

Now, we give the following example.

Example 9 Let X=[0,1] with the usual metric and usual order ≤. We define a mapping T:X→X by

$$Tx=\left\{ \begin{array}{cc} 0 & ,\text{if}\;x\in [0,\frac{1}{5}],\\ \frac{1}{5} & ,\text{if}\;x\in (\frac{1}{5},1]. \end{array}\right.$$

It can be easily seen that *T* is discontinuous and does not satisfy (6) for any α,β,γ∈[0,1) with 0≤α+2β+γ<1 when $x=\frac{1}{5},y=1$. Now $T^{2}(x)=0$ for all x∈[0,1). It can be verified that $T^{2}$ satisfies the condition of Theorem 13 and 0 is a fixed point of $T^{2}$.

Remark 1 In [13], instead of condition (3), the authors use the following weaker condition:

(13) $$\text{if a nondecreasing (nonincreasing) sequence}\;\{x_{n}\}\to x\;\text{in}\;X,\;\text{then}\;\;\;\;\;\;\;\;\;\;\;x_{n}\leq x\;(x\leq x_{n}),\;\text{for all}\;n\in N,$$

we have not been able to prove Theorem 2, Theorem 8 and its consequences using (13).

Some other consequences of the main Theorem 8 for the self mapping involving in the integral type contractions are as follows.

Corollary 4 *Let* (X,d,⪯) *be a complete partially ordered metric space. Suppose that a nondecreasing, continuous self mapping T on X satisfies the following condition*

(14) $$\begin{array}{cc} \int_{0}^{d(Tx,Ty)}d\psi & \leq \alpha \int_{0}^{\frac{d(x,Tx)d(y,Ty)}{d(x,y)}}d\psi +\beta \int_{0}^{d(x,Ty)+d(y,Tx)}d\psi +\gamma \int_{0}^{d(x,y)}d\psi \\ & \;\;\;+L\int_{0}^{\min\{d(x,Ty),d(y,Tx),d(x,Tx)\}}d\psi , \end{array}$$

*for all distinct* x,y∈X *with* x⪯y *and, there exist* α,β,γ∈[0,1) *with* 0≤α+2β+γ<1 *and* L≥0*. If there exists* $x_{0}\in X$ *such that* $x_{0}⪯Tx_{0}$*, then T has at least one fixed point in X.*

Similarly, the following result is the consequence of Corollary 1.

Corollary 5 *Let* (X,d,⪯) *be a complete partially ordered metric space. Suppose that a nondecreasing, continuous self mapping T on X satisfies the following condition*

(15) $$\int_{0}^{d(Tx,Ty)}d\psi \leq \alpha \int_{0}^{\frac{d(x,Tx)d(y,Ty)}{d(x,y)}}d\psi +\beta \int_{0}^{d(x,Ty)+d(y,Tx)}d\psi +\gamma \int_{0}^{d(x,y)}d\psi ,$$

*for all distinct* x,y∈X *with* x⪯y *and, there are some* α,β,γ∈[0,1) *such that* 0≤α+2β+γ<1*. If there exists* $x_{0}\in X$ *such that* $x_{0}⪯Tx_{0}$*, then T has a fixed point in X.*

## Declarations

### Author contribution statement

N. Seshagiri Rao: Conceived and designed the analysis; Analyzed and interpreted the data; Contributed analysis tools or data; Wrote the paper.

K. Kalyani: Conceived and designed the analysis; Analyzed and interpreted the data; Wrote the paper.

### Funding statement

This research did not receive any specific grant from funding agencies in the public, commercial, or not-for-profit sectors.

### Data availability statement

Data will be made available on request.

### Declaration of interests statement

The authors declare no conflict of interest.

### Additional information

No additional information is available for this paper.
